# Prevalence of lung cancer in chronic obstructive pulmonary disease: A systematic review and meta-analysis

**DOI:** 10.3389/fonc.2022.947981

**Published:** 2022-09-16

**Authors:** Guixiang Zhao, Xuanlin Li, Siyuan Lei, Hulei Zhao, Hailong Zhang, Jiansheng Li

**Affiliations:** ^1^ Co-Construction Collaborative Innovation Center for Chinese Medicine and Respiratory Diseases by Henan and Education Ministry of P.R. China, Henan University of Chinese Medicine, Zhengzhou, China; ^2^ Henan Key Laboratory of Chinese Medicine for Respiratory Disease, Henan University of Chinese Medicine, Zhengzhou, China; ^3^ Department of Respiratory Diseases, The First Affiliated Hospital of Henan University of Chinese Medicine, Zhengzhou, China

**Keywords:** chronic obstructive pulmonary disease, lung cancer, prevalence, meta-analysis, systematic review

## Abstract

**Background:**

There is growing evidence that chronic obstructive pulmonary disease (COPD) can increase the risk of lung cancer, which poses a serious threat to treatment and management. Therefore, we performed a meta-analysis of lung cancer prevalence in patients with COPD with the aim of providing better prevention and management strategies.

**Methods:**

We systematically searched PubMed, EMBASE, Web of Science, and Cochrane Library databases from their inception to 20 March 2022 to collect studies on the prevalence of lung cancer in patients with COPD. We evaluated the methodological quality of the included studies using the tool for assessing the risk of bias in prevalence studies. Meta-analysis was used to determine the prevalence and risk factors for lung cancer in COPD. Subgroup and sensitivity analyses were conducted to explore the data heterogeneity. Funnel plots combined with Egger’s test were used to detect the publication biases.

**Results:**

Thirty-one studies, covering 829,490 individuals, were included to investigate the prevalence of lung cancer in patients with COPD. Pooled analysis demonstrated that the prevalence of lung cancer in patients with COPD was 5.08% (95% confidence interval [CI]: 4.17–6.00%). Subgroup analysis showed that the prevalence was 5.09% (95% CI: 3.48–6.70%) in male and 2.52% (95% CI: 1.57–4.05%) in female. The prevalence of lung cancer in patients with COPD who were current and former smokers was as high as 8.98% (95% CI: 4.61–13.35%) and 3.42% (95% CI: 1.51–5.32%); the incidence rates in patients with moderate and severe COPD were 6.67% (95% CI: 3.20–10.14%) and 5.57% (95% CI: 1.89–16.39%), respectively, which were higher than the 3.89% (95% CI: 2.14–7.06%) estimated in patients with mild COPD. Among the types of lung cancer, adenocarcinoma and squamous cell carcinoma were the most common, with incidence rates of 1.59% (95% CI: 0.23–2.94%) and 1.35% (95% CI: 0.57–3.23%), respectively. There were also differences in regional distribution, with the highest prevalence in the Western Pacific region at 7.78% (95% CI: 5.06–10.5%), followed by the Americas at 3.25% (95% CI: 0.88–5.61%) and Europe at 3.21% (95% CI: 2.36–4.06%).

**Conclusions:**

This meta-analysis shows that patients with COPD have a higher risk of developing lung cancer than those without COPD. More attention should be given to this result in order to reduce the risk of lung cancer in these patients with appropriate management and prevention.

**Systematic review registration:**

International prospective register of systematic reviews, identifier CRD42022331872.

## Introduction

Chronic obstructive pulmonary disease (COPD) is a common respiratory disease characterized by persistent respiratory symptoms and airflow restriction, with a high risk of morbidity, disability rate, mortality, and heavy disease burden, which seriously impacts human health (1, 2). The prevalence of COPD has increased by 44.2% and reached 174.5 million individuals worldwide from 1990 to 2015 (3), although it remains so far underestimated (4). More than 5.4 million people will die from COPD and related diseases each year by 2060, according to predictions made by the World Health Organization (WHO) (5). As the third leading cause of death worldwide (6, 7), COPD has caused serious economic burden and social pressure and has become a major public health problem (8, 9). The cost of treating COPD is expected to be $800.09 billion in the next 20 years, which is approximately $40 billion per year in the United States (10). Patients with COPD are at a high risk of multiple comorbidities, which have a significant impact on disease progression, hospitalization, and mortality (11–13). As reported by the National Lung Screening Trial, the incidence of lung cancer in patients with airway obstruction has increased by 2.15 times (14), and lung cancer is a critical cause of hospitalization and death in patients with COPD (15). Therefore, it is necessary to emphasize the importance of prevention and treatment of lung cancer in patients with COPD.

Lung cancer is one of the malignant tumors with the highest morbidity and mortality worldwide, especially in male patients, and has a devastating impact on the life expectancy (16). The number of individuals newly diagnosed with lung cancer was up to 2.2 million (11.4%) as reported by Global Cancer Statistics in 2020, and the number of patients with lung cancer that died in the world that year was approximately 1.8 million (18.0%) (16). The onset of lung cancer is insidious, and 75% of patients have reached an advanced stage when visiting a doctor (17). Related studies have shown that the 5-year survival rate of patients with advanced lung cancer is less than 5% (18). Importantly, it has been reported that 45–63% of patients with lung cancer are globally affected by COPD (19). As two major respiratory diseases with the highest mortality, patients with COPD seem to have a higher incidence of lung cancer than patients without COPD (20, 21).

Although previous studies have found that COPD increases the risk of lung cancer, no unified conclusions have been reached owning to the differences in survey periods, sample demographic characteristics, and types of included studies. Currently, there is still no specific epidemiological conclusion concerning an evaluation of the risk of lung cancer in patients with COPD, according to the Global Initiative for Chronic Obstructive Lung Disease (GOLD) guidelines. In addition, several high-quality observational studies (22–29) investigating the risk of lung cancer in patients with COPD have recently been published. Therefore, we systematically collected data from existing observational population-based studies to determine whether patients with COPD have an increased risk of lung cancer.

## Methods

This study was performed in strict accordance with the Preferred Reporting Items for Systematic Reviews and Meta-Analyses (PRISMA 2020) (30). The protocol of this systematic review was registered in the Prospective Register of Systematic Reviews (PROSPERO), and the registry number is CRD42022331872.

### Search strategy

We systematically searched the PubMed, EMBASE, Web of Science, and Cochrane Library databases without language restrictions from their inception to 20 March 2022. Medical Subject Headings (MESH) terms and keywords used in the search were (“Pulmonary Disease, Chronic Obstructive” OR “Chronic Obstructive Pulmonary Disease*” OR “Chronic Airflow Obstruction*” OR “Chronic Obstructive lung Disease” OR “COPD” OR “COAD” OR “Chronic Obstructive Airway Disease”) AND (“lung neoplasm*” OR “pulmonary neoplasm*” OR “lung cancer” OR “pulmonary cancer” OR “lung tumor” OR “pulmonary tumor” OR “lung carcinoma”). Detailed retrieval strategies and steps are presented in **Supplement Table 1**. Furthermore, the reference lists of the retrieved articles and relevant reviews were also manually examined to identify other potentially eligible studies.

### Eligibility criteria

Articles were included if they met the following criteria: (1) observational studies that reported on the prevalence of lung cancer in patients with COPD; (2) the exposed group consisted of patients with any grade of COPD, and the control group consisted of patients without COPD; (3) the prevalence of lung cancer was chosen as the primary outcome.

### Exclusion criteria

(1) Conference abstracts or study protocols; (2) duplicate published studies based on the same observation population; and (3) containing data with errors and patients diagnosed with non-COPD upon our failure to extract information.

### Study selection

The study selection was conducted independently by two reviewers (GX Zhao and XL Li) to screen suitable articles. Duplicate and irrelevant studies were excluded based on their titles and abstracts. Thereafter, the full text of each potentially eligible study was carefully read and reviewed based on the inclusion and exclusion criteria stated above. Any disagreements were resolved by consultation with a third investigator (JS Li) until consensus was reached.

### Data extraction

Two reviewers (GX Zhao and SY Lei) independently followed the data extraction guidelines for systematic evaluation and meta-analysis (31), using predesigned forms to extract and summarize the relevant information of the eligible studies. The following information was extracted: Author, year of publication, study type, country, study period or year of follow-up, sample size, lung cancer diagnosis, sex distribution, mean or median age, COPD severity, smoking status, lung cancer type, and confounder adjustment. Any disagreements were resolved with a third investigator (HL Zhang) through consultation until a consensus was reached.

### Assessment of risk of bias

We used the disease prevalence quality tool modified by Hoy et al. (32) to assess the quality of the included studies, which consisted of 10 items. The score of each item was 1 or 0, and the total scores of each observational study was between 0 and 10, with higher scores indicating better study quality. Study quality was defined according to the total score of each study, with scores of 0–5, 6–8, and 9–10 for low, moderate, and high quality, respectively.

### Statistical analysis

Data were extracted from the included studies to calculate the prevalence of lung cancer in patients with COPD. We performed a meta-analysis using the double arcsine transformation of proportions, which is appropriate for binomial data and allows the adoption of inverse variance methods to calculate binomial and test score-based CIs (33). The chi-square test and *I* ^2^ value were used to assess the heterogeneity. A high heterogeneity was existed if *P* < 0.1 or *I* ^2^ > 50%, and the random-effects model was adopted. Subgroup analysis was conducted to determine whether the prevalence was influenced by sex, smoking status, COPD severity, cancer type, and region. Otherwise, a fixed-effects model was selected. To confirm the robustness of the overall results, we performed a sensitivity analysis by excluding one study each time and then re-running it. Funnel plots was used to visually detect publication bias, and Egger’s regression test was used to statistically inspect publication bias. We pooled OR and 95% CIs to assess whether sex, COPD severity, and smoking status were risk factors for lung cancer in patients with COPD. All statistical analyses were conducted by using Stata statistical software version 15.1.

## Results

### Identification of studies

**Figure 1:**
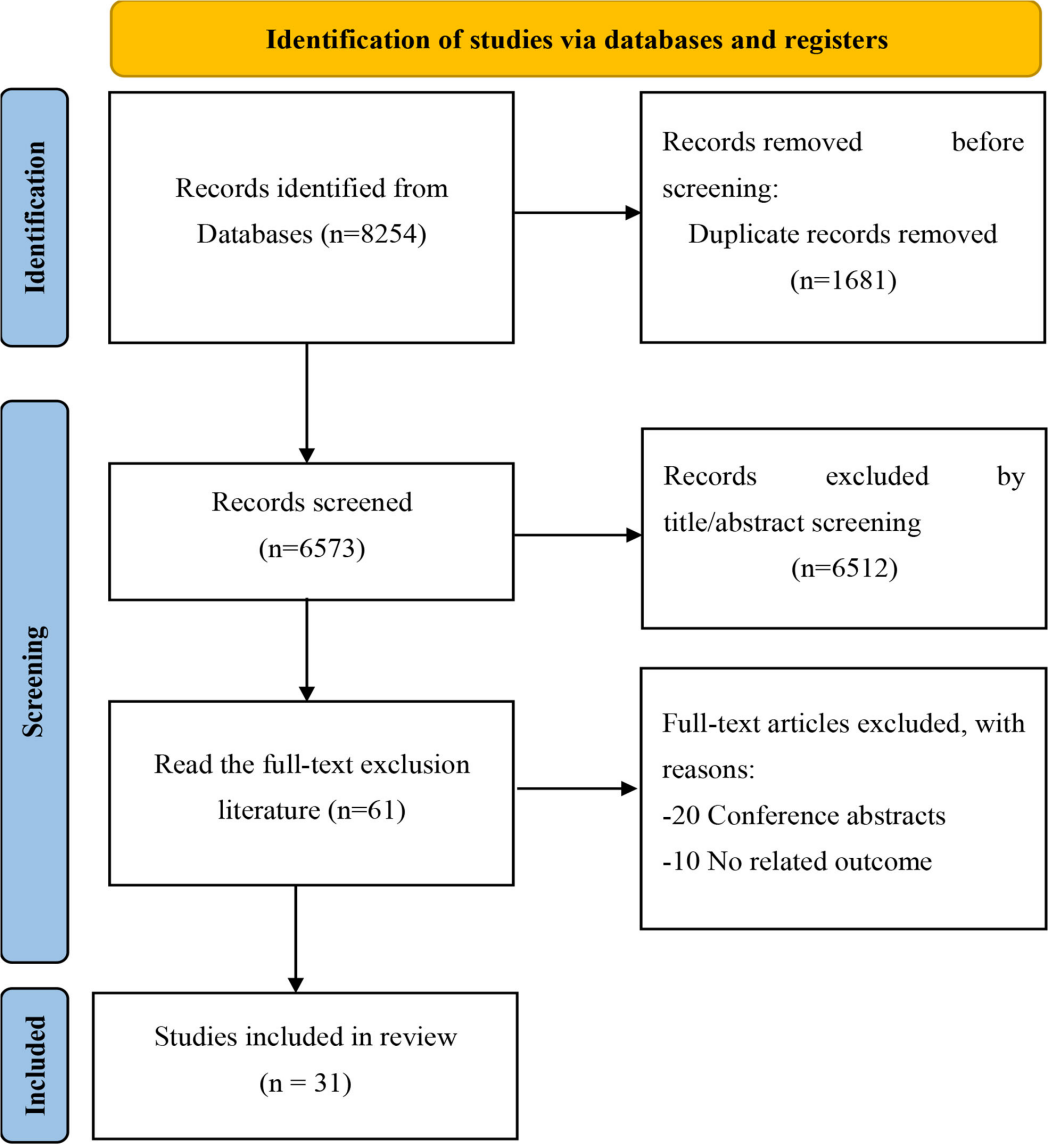
Flowchart of study identification for meta-analysis.

A total of 8,254 related studies were retrieved through electronic and manual searching from the initial examination, of which 1,681 duplicates, 6,573 unrelated studies were excluded after reading titles and abstracts. After screening qualified articles by reading the full text, 31 (13, 22–26, 34–55) studies were included in the meta-analysis. The study selection process is shown in (**Figure 1**).

### Study characteristics

Overall, we included 31studies covering 829,490 patients with COPD, including twenty-one (13, 22–26, 34–48) cohort studies, three (27, 49, 50) case-control studies and seven (28, 29, 51–55) cross-sectional studies. These studies were published from 2003 to 2022 with definite diagnostic criteria, and the sample sizes ranged from 198 to 236,494. Data were acquired from 13 countries: China, Korea, Japan, the United States, the United Kingdom, Germany, Denmark, Norway, Spain, Lithuania, Sweden, Turkey, and the Netherlands. Fifteen, twelve, and two studies were conducted in the European region, Western Pacific region, and Americas, respectively. Besides, two studies (13, 41) included both the United States and Spain, simultaneously. The main characteristics of the included trials are summarized in **Table 1**.

**Table 1:** Basic characteristics of the included studies.

Reference	Country	Study design	COPD				Lung cancer			Duration or range of follow-up, years
			Diagnosis	Sample size	Age (years)	M/F	Diagnosis	Sample size	M/F
Sandelin et al.2018 (22)	Swedish	Retrospective cohort	ICD-10-CM code J44	19894	–	9452/110442	ICD-10 code C34	594	291/303	1999.1.1-2009.12.31
Ahn et al., 2020 (23)	Korean	Retrospective cohort	ICD-10 codes J43-J44	11551	–	6172/5379	ICD-10 codes C33-C34	1136	–	2004.1.1-2015.12.31
Husebøet al., 2019 (24)	Norway	Prospective cohort	Clinical and Spirometry confirmed	433	63.5 ± 6.9	258/175	Norwegian Cancer Registry	28	–	9
Park et al., 2020 (25)	Korean	Retrospective cohort	ICD-10 codes J43-J44	58972	–	–	ICD-10 code C33 or C34	290	–	2002.1.1-2013.12.31
Machida et al., 2021 (26)	Japan	Prospective cohort	Spirometry confirmed	224	70.4 ± 8.4	214/10	CT	19	19	2014.1-2020.4
Sakai et al., 2020 (27)	Japan	Retrospective cohort	Spirometry confirmed	198	69.7 ± 8.0	184/14	–	43	–	2011.4.1-2015.7.16
Montserrat et al., 2021 (28)	Spain	Retrospective cross-sectional	Spirometry confirmed	24135	72 ± 11	18612/5523	ICD-10	552	–	2012.1.1-2017.12.31
Jurevičienė et al., 2022 (29)	Lithuanian	Retrospective cross-sectional	ICD-10-AMD J44.8	4834	67.2 ± 8.4	3338/1496	ICD 10 code C33, C34	186	–	2012.1.1-2014.6.30
Thomsen et al., 2012 (34)	Denmark	Prospective cohort	ICD8: 490–492; ICD10: J44	8656	65 (57, 74)	47%/53%	ICD10 code C34	93	–	5
Chubachi et al., 2016 (35)	Japan	Prospective cohort	Spirometry confirmed	311	72.3 ± 8.2	278/33	clinical history and medical records	13	–	2
Divo et al., 2012 (13)	USA + Spain	Prospective cohort	Spirometry confirmed	1659	66 ± 9	1477/182	medical record and direct questioning	151	–	1997.11-2010.3
Westerik et al., 2017 (36)	Dutch	Retrospective cohort	ICPC code R95 in the electronic medical record	14603	66.5 ± 11.5	7749/6854	ICPC code R84	317	–	2012–2013.12.31
Lin et al.2013 (37)	China	Retrospective case-control	ICD-9-CM code 496	2630	–	2096/534	cytologically or histologically confirmed	181	–	2006.1.1-2011.12.31
de Torres et al., 2007 (38)	Spain	Prospective cohort	Spirometry confirmed	1166	54 ± 8	74% vs 26%	CT and Biopsy	23	–	2000.9-2005.12
Purdue et al., 2007 (39)	Swedish	Retrospective cohort	Spirometry confirmed	6849	–	6849	ICD-7 codes 162, 163	175	175	1971-2001
Wilson et al., 2008 (40)	USA	Prospective cohort	Spirometry confirmed	1486	–	–	medical records and pathology reports	67	–	3.26
Rodríguez et al., 2010 (41)	UK	Prospective cohort	Oxford Medical Information System [OXMIS] and Read codes	1924	–	–	Oxford Medical Information System [OXMIS] and Read codes	48	–	1996.1.31-2001
de Torres et al., 2011 (42)	USA + Spain	Prospective cohort	Spirometry confirmed	2507	65 ± 9	2307/200	medical records and pathology reports	215	205/10	1997.1-2009.12
Kornum et al., 2012 (43)	Danish	Prospective cohort	ICD-8 codes:491-492; ICD-10 codes: J41-J44	236494	–	129344/107150	medical records and pathology reports	10118	–	1980-2008
Shen et al., 2014 (44)	China	Retrospective cohort	ICD-9-CM 491, 492, and 496	20730	70	13291/7439	ICD-9-CM 162	729	575/154	1998-2011
Hasegawa et al., 2014 (45)	Japan	Retrospective cohort	ICD-10 codes: J41, J42, J43, J44	172707	–	136632/36075	ICD-10 codes C34	13930	–	2010.7.1-2013.3.31
Roberts et al., 2011 (46)	UK	Prospective cohort	ICD10 code J44 and J45/46 (asthma) later confirmed as COPD	9716	73 ± 10	4906/4810	Medical records confirmed by physician	180	–	2008.3-2008.8
Ställberg et al., 2018 (47)	Swedish	Retrospective cohort	ICD-10 code: J44	17479	–	–	ICD-10 code: C34	1091	–	2000-2014
Mannino et al., 2003 (48)	USA	Prospective cohort	Spirometry confirmed	5402	–	2473/2929	ICD-9 code: 162	113	–	1971-1992
Schneider et al., 2010 (49)	UK	Retrospective case-control	OXMIS codes	35772	–	18351/17421	OXMIS codes	2585	1526/1059	1995.1.1-2005.12.31
Greulich et al., 2017 (50)	Germany	Retrospective case-control	ICD-10: J41, J43, J44	146141	67.2 ± 12.41	51%/49%	ICD-10 code not provided	2663	–	2013.1.1-2014.12.31
Jo et al., 2015 (51)	Korean	Retrospective cross-sectional	ICD-10 code: J44	744	65.0 ± 9.40		ICD-10 code: C34	97	–	2010-2012
Deniz et al., 2016 (52)	Turkey	Retrospective cross-sectional	Spirometry confirmed	3095	71.9 ± 10.5	2434/661	Medical records	58	–	2014.1.1-2014.12.31
Jung et al., 2018 (53)	Korean	Retrospective cross-sectional	ICD 10 code J44	15949	69 (60, 76)	9039/6910	ICD 10 code C34	753	590/163	2011.1-2011.12
Masuda et al., 2017 (54)	Japan	Retrospective cohort	Spirometry confirmed	920	–	651/269	self-reported and confirmed by a physician	13	10/3	2009.4-2010.3
Nishida et al., 2017 (55)	Japan	Retrospective cross-sectional	Spirometry confirmed	2309	69.06 ± 10.53	1549/760	ICD-10 code C34	354	–	2005.9-2008.12

COPD, chronic obstructive pulmonary disease; F: female; M: male; ICD, International Classification of Diseases; -: No mentioned.

### Quality assessment

We evaluated the quality of the included studies, and the average score of the included cohort studies, case-control studies, and cross-sectional studies were 7.90, 8.33, and 8.43, respectively, which suggested that the studies included in our meta-analysis were of high quality. Ten cohort studies (22, 23, 25, 34, 36, 41, 43 ^-^ 45, 48), two case-control studies (49, 50) and five cross-sectional studies (28, 37, 51, 53, 54) with scores ≥ 9 were classified as high-quality studies, and the remaining observational studies were of moderate quality. The specific score information for all included observational studies is shown in **Table 2**.

**Table 2:** Risk of bias for included studies.

Study Items	1	2	3	4	5	6	7	8	9	10	Scores	Overall of quality
**Cohort studies**												
Thomsen, M. 2012 (34)	1	0	1	1	1	1	1	1	1	1	9	High
S. Chubachi, 2016 (35)	0	0	0	1	1	1	1	1	1	1	7	Moderate
M. Divo, 2012 (13)	0	0	0	1	1	1	1	1	1	1	7	Moderate
J.A.M. Westerik, 2017 (36)	1	0	1	1	1	1	1	1	1	1	9	High
Lin, S. H. 2013 (37)	0	0	0	1	1	1	1	1	1	1	7	Moderate
Sandelin, M. 2018 (22)	1	0	1	1	1	1	1	1	1	1	9	High
Ahn, S. V. 2020 (23)	1	0	1	1	1	1	1	1	1	1	9	High
de Torres, J. P. 2007 (38)	0	0	0	1	1	1	1	1	1	1	7	Moderate
Purdue, M. P. 2007 (39)	0	0	0	1	1	1	1	1	1	1	7	Moderate
Wilson, D. O. 2008 (40)	0	0	0	1	1	1	1	1	1	1	7	Moderate
Rodríguez, L. A. 2010 (41)	1	0	1	1	1	1	1	1	1	1	9	High
De Torres, J. P. 2011 (42)	0	0	0	1	1	1	1	1	1	1	7	Moderate
Kornum, J. B. 2012 (43)	1	0	1	1	1	1	1	1	1	1	9	High
Shen, T. C. 2014 (44)	1	0	1	1	1	1	1	1	1	1	9	High
Husebø, G. R. 2019 (24)	0	0	0	1	1	1	1	1	1	1	7	Moderate
Park, H. Y. 2020 (25)	1	0	1	1	1	1	1	1	1	1	9	High
Machida, H. 2021 (26)	0	0	0	1	1	1	0	1	1	1	6	Moderate
W. Hasegawa, 2014 (45)	1	0	1	1	1	1	1	1	1	1	9	Moderate
C.M. Roberts, 2011 (46)	0	0	0	1	1	1	1	1	1	1	7	Moderate
Ställberg, B. 2018 (47)	0	0	0	1	1	1	1	1	1	1	7	Moderate
Mannino DM, 2003 (48)	1	0	1	1	1	1	1	1	1	1	9	High
**Case-control studies**												
Schneider, C. 2010 (49)	1	0	1	1	1	1	1	1	1	1	9	High
Greulich, T. 2017 (50)	1	0	1	1	1	1	1	1	1	1	9	High
Sakai, T. 2020 (27)	0	0	0	1	1	1	1	1	1	1	7	Moderate
**Cross-sectional studies**												
Y.S. Jo, 2015 (51)	1	0	1	1	1	1	1	1	1	1	9	High
S. Deniz, A. 2016 (52)	0	0	0	1	1	1	1	1	1	1	7	Moderate
Jung, H. I. 2018 (53)	1	0	1	1	1	1	1	1	1	1	9	High
Montserrat-Capdevila, J. 2021 (28)	1	0	1	1	1	1	1	1	1	1	9	High
Jurevičienė, E. 2022 (29)	1	0	1	1	1	1	1	1	1	1	9	High
Masuda, S. 2017 (54)	1	0	1	1	1	1	1	1	1	1	9	High
Nishida, Y. 2017 (55)	0	0	0	1	1	1	1	1	1	1	7	Moderate

1.Was the study’s target population a close representation of the national population in relation to relevant variables?

2.Was the sampling frame a true or close representation of the target population?

3.Was some form of random selection used to select the sample, or was a census undertaken?

4.Was the likelihood of nonresponse bias minimal?

5.Were data collected directly from the subjects (as opposed to a proxy)?

6.Was an acceptable case definition used in the study?

7.Was the study instrument that measured the parameter of interest shown to have validity and reliability?

8.Was the same mode of data collection used for all subjects?

9.Was the length of the shortest prevalence period for the parameter of interest appropriate?

10.Were the numerator(s) and denominator(s) for the parameter of interest appropriate?

### Prevalence of lung cancer in COPD patients

**Figure 2:**
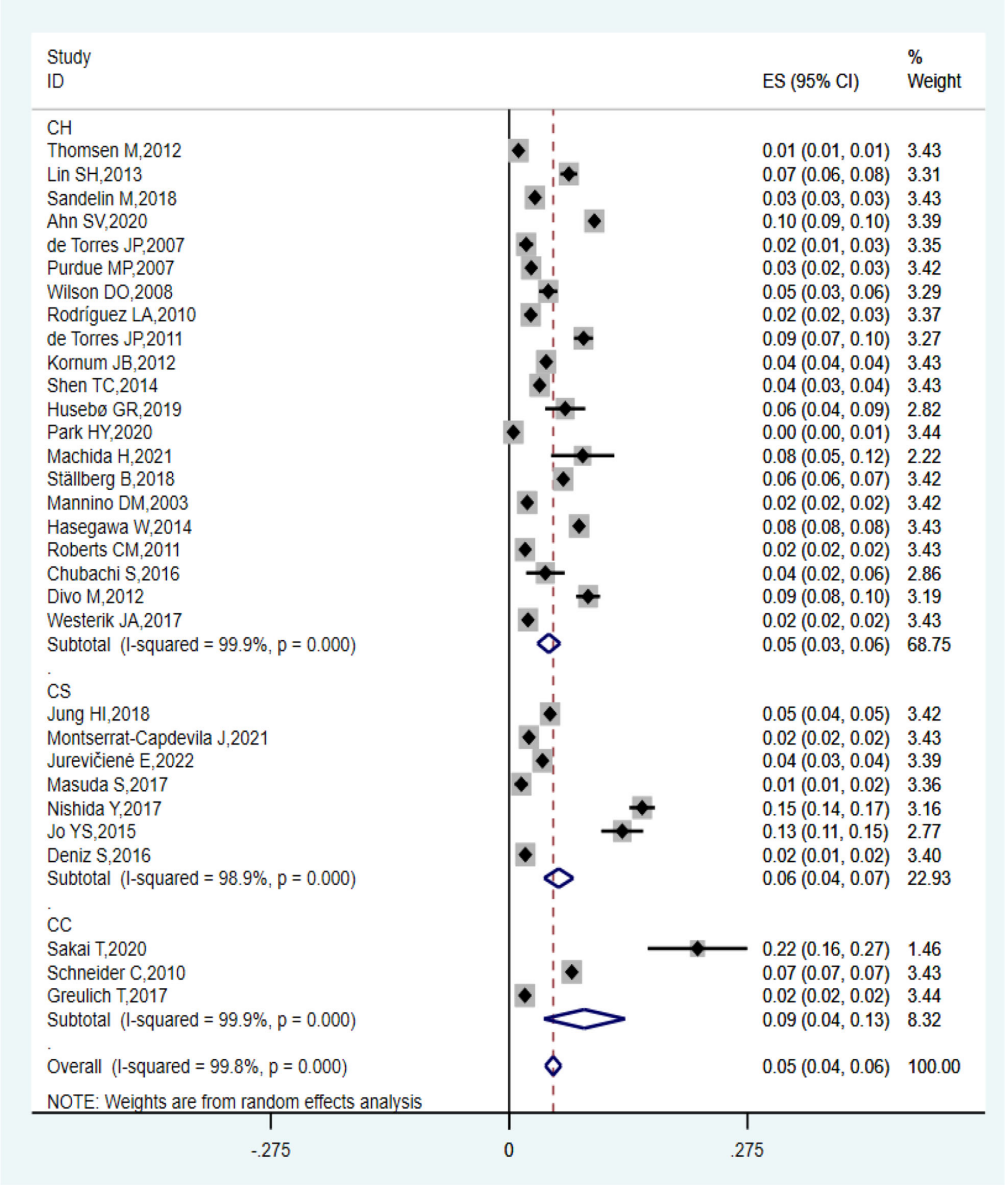
Forest plot showing the prevalence of lung cancer in COPD.

Thirty-one observational studies reported that the prevalence of lung cancer among patients with COPD ranged from 0.49% to 21.7%, and the overall estimated prevalence was 5.08% (95% CI: 4.17–6.00%; *I* ^2^ = 99.8%, *P* = 0.000). Of these studies, the estimated pooled prevalence of 21 cohort studies, three case-control studies, and seven cross-sectional studies were 4.58% (95% CI: 3.27–5.89%; *I* ^2^ = 99.9%, *P* = 0.000), 8.67% (95% CI: 4.00–13.35%; *I* ^2^ = 99.9%, *P* = 0.000), and 5.72% (95% CI: 4.02–7.41%; *I* ^2^ = 98.9%, *P* = 0.000), respectively, as shown in **Figure 2**. Sensitivity analysis proved that the estimated pooled prevalence was still ≥ 4% after excluding one study at a time, which confirmed the high stability of our results in **Table 3**.

**Table 3:** Sensitivity analysis showing the effect of lung cancer in COPD.

Study design	Deletion	Result
Cohort study	Thomsen M, 2012 (34)	ES = 5.23%, 95% CI [4.29%, 6.18%]
	Lin SH, 2013 (37)	ES = 5.02%, 95% CI [4.09%, 5.95%]
	Sandelin M, 2018 (23)	ES = 5.17%, 95% CI [4.22%, 6.11%]
	Ahn SV, 2020 (24)	ES = 4.91%, 95% CI [3.99%, 5.82%]
	de Torres JP, 2007 (38)	ES = 5.19%, 95% CI [4.26%, 6.12%]
	Purdue MP, 2007 (39)	ES = 5.18%, 95% CI [4.24%, 6.11%]
	Wilson DO, 2008 (40)	ES = 5.10%, 95% CI [4.17%, 6.04%]
	Rodríguez, L. A. 2010 (41)	ES = 5.18%, 95% CI [4.24%, 6.11%]
	de Torres JP, 2011 (42)	ES = 4.97%, 95% CI [4.04%, 5.89%]
	Kornum JB, 2012 (43)	ES = 5.14%, 95% CI [4.16%, 6.12%]
	Shen TC, 2014 (44)	ES = 5.15%, 95% CI [4.21%, 6.09%]
	Husebø GR, 2019 (24)	ES = 5.04%, 95% CI [4.12%, 5.97%]
	Park HY, 2020 (25)	ES = 5.22%, 95% CI [4.33%, 6.11%]
	Machida H, 2021 (26)	ES = 5.01%, 95% CI [4.08%, 5.93%]
	Ställberg B, 2018 (47)	ES = 5.04%, 95% CI [4.12%, 5.97%]
	Mannino DM, 2003 (48)	ES = 5.19%, 95% CI [4.26%, 6.13%]
	Hasegawa W, 2014 (45)	ES = 4.85%, 95% CI [4.10%, 5.59%]
	Roberts CM, 2011	ES = 5.20%, 95% CI [4.26%, 6.15%]
	Chubachi S, 2016 (35)	ES = 5.11%, 95% CI [4.18%, 6.04%]
	Divo M, 2012 (13)	ES = 4.95%, 95% CI [4.02%, 5.88%]
	Westerik JA, 2017 (36)	ES = 5.20%, 95% CI [4.25%, 6.14%]
Cross-sectional study	Jung, HI, 2018 (53)	ES = 5.10%, 95% CI [4.17%, 6.03%]
	Montserrat-Capdevila J. 2021 (28)	ES = 5.20%, 95% CI [4.24%, 6.15%]
	Jurevičienė E. 2022 (29)	ES = 5.13%, 95% CI [4.20%, 6.06%]
	Masuda S, 2017 (54)	ES = 5.21%, 95% CI [4.28%, 6.14%]
	Nishida Y, 2017 (55)	ES = 4.75%, 95% CI [3.82%, 5.67%]
	Jo YS, 2015 (51)	ES = 4.86%, 95% CI [3.93%, 5.78%]
	Deniz S, 2016 (52)	ES = 5.20%, 95% CI [4.27%, 6.13%]
Case-control study	Sakai T. 2020 (27)	ES = 4.84%, 95% CI [3.92%, 5.76%]
	Schneider C, 2010 (49)	ES = 5.00%, 95% CI [4.09%, 5.91%]
	Greulich T, 2017 (50)	ES = 5.27%, 95% CI [4.20%, 6.35%]

### Subgroup analysis

In terms of sex, nine studies (22, 26, 39, 41, 44, 48, 49, 54, 54) investigated the prevalence of lung cancer in male patients with COPD, covering 62,627 individuals, with a prevalence ranging from 1.54% to 8.89%, and the estimated pooled prevalence was 5.09% (95% CI: 3.48–6.70%; *I* ^2^ = 98.8%, *P* = 0.000). Eight (22, 26, 41, 44, 48, 49, 53, 54) studies illustrated that the prevalence of lung cancer in female patients with COPD was 2.52% (95% CI: 1.57–4.05%; *I* ^2^ = 99.9%, *P* = 0.000) (**Table 4**).

**Table 4:** Subgroup analysis of the prevalence of lung cancer in COPD.

Subgroups	Studies	Total	Events	Model	ES	Heterogeneity		P _difference_
	*n*				(95%CI)	*I* ^2^	*P*				
Gender								
Male	9	62627	3472	random	5.09% (3.48%, 6.70%)	98.80%	0	0.000
Female	8	45620	1724	random	2.52% (1.57%, 4.05%)	99.90%	0	0.000
Smoking status								
Never smoking	4	52863	744	random	0.68% (0.10%, 4.65%)	100%	0	0.000
Former smoking	4	20812	323	random	3.42% (1.51%, 5.32%)	97.600%	0	0.000
Current smoking	5	9879	731	random	8.98% (4.61%, 13.35%)	98.40%	0	0.000
COPD severity								
Mild	6	5311	151	random	3.89% (2.14%, 7.06%)	99.40%	0	0.000
Moderate	3	1986	141	random	6.67% (3.20%, 10.14%)	87.00%	0	0.000
Severe	2	835	70	random	5.57% (1.89%, 16.39%)	94.70%	0	0.000
Cancer type								
Small cell carcinoma	3	8213	35	random	0.78% (0.78%, 1.77%)	99.70%	0	0.000
Adenocarcinoma	3	8213	68	random	1.59% (0.23%, 2.94%)	90.90%	0	0.022
Squamous cell carcinoma	3	8213	75	random	1.35% (0.57%, 3.23%)	99.70%	0	0.000
Region								
European	15	531191	18711	random	3.21% (2.36%, 4.06%)	99.6%	0	0.000
Western Pacific region	12	287245	17558	random	7.78% (5.06%, 10.5%)	99.9%	0	0.000
Americas	2	6888	180	random	3.25% (0.88%, 5.61%)	94.40%	0	0.007

CH, Cohort study; CS, Cross-sectional study; CC, Case-control study.

Six studies comprehensively described the influence of smoking status on the prevalence of lung cancer in patients with COPD. Of these studies, the prevalence of lung cancer was estimated in current smokers in five (23, 26, 39, 42, 48), in former smokers in four (23, 25, 39, 48), and in never smokers in four (23, 25, 39, 48). The estimated prevalence according to the smoking status was 8.98% (95% CI: 4.61–13.35%; *I* ^2^ = 98.4%, *P* = 0.000), 3.42% (95% CI: 1.51–5.32%; *I* ^2^ = 97.6%, *P* = 0.000), and 0.68% (95% CI: 0.10–4.65%; *I* ^2^ = 100%, *P* = 0.000), respectively (**Table 4**).

Regarding the severity of COPD, six studies (26, 39, 40, 42, 48, 54) provided comprehensive information on the incidence of COPD combined with lung cancer at different stages. Among them, six (26, 39, 40, 42, 48, 54), three (26, 40, 42), and two (26, 42) studies reported lung cancer prevalence in patients with mild, moderate, and severe COPD, respectively, with a pooled prevalence of 3.89% (95% CI: 2.14–7.06%; *I* ^2^ = 99.4%, *P* = 0.000), 6.67% (95% CI: 3.20–10.14%; *I* ^2^ = 87%, *P* = 0.000), and 5.57% (95% CI: 1.89–16.39%; *I* ^2^ = 94.7%, *P* = 0.000), respectively (**Table 4**).

With respect to the histological subtype of lung cancer, three studies (27, 38, 39) described specific categories and the overall pooled prevalence of small cell lung cancer, adenocarcinoma, and squamous cell carcinoma in patients with COPD was 0.78% (95% CI: 0.34–1.77%; *I* ^2^ = 99.7%, *P* = 0.000), 1.59% (95% CI: 0.23–2.94%; *I* ^2^ = 90.9%, *P* = 0.022) and 1.35% (95% CI: 0.57–3.23%; *I* ^2^ = 99.7%, *P* = 0.000), respectively (**Table 4**).

The prevalence of lung cancer among patients with COPD in different regions is of great significance. Fifteen (22, 24, 28, 29, 34, 36, 38, 39, 41, 43, 46, 47, 49, 50, 52) studies reported lung cancer prevalence in patients with COPD in the European region, ranging from 1.07% to 7.23%, with an estimated prevalence of 3.21% (95% CI: 2.36–4.06%; *I* ^2^ = 99.6%, *P* = 0.000). In addition, 12 (23, 25–27, 35, 37, 44, 45, 51, 53–55) and two (40, 48) studies reported that the prevalence of lung cancer in patients with COPD in the Western Pacific and the Americas region, with a pooled prevalence of 7.78% (95% CI: 5.06–10.5%; *I* ^2^ = 99.9%, *P* = 0.000) and 3.25% (95% CI: 0.88–5.61%; *I* ^2^ = 94.4%, *P* = 0.007), respectively (**Table 4**).

### Publication bias

**Figure 3:**
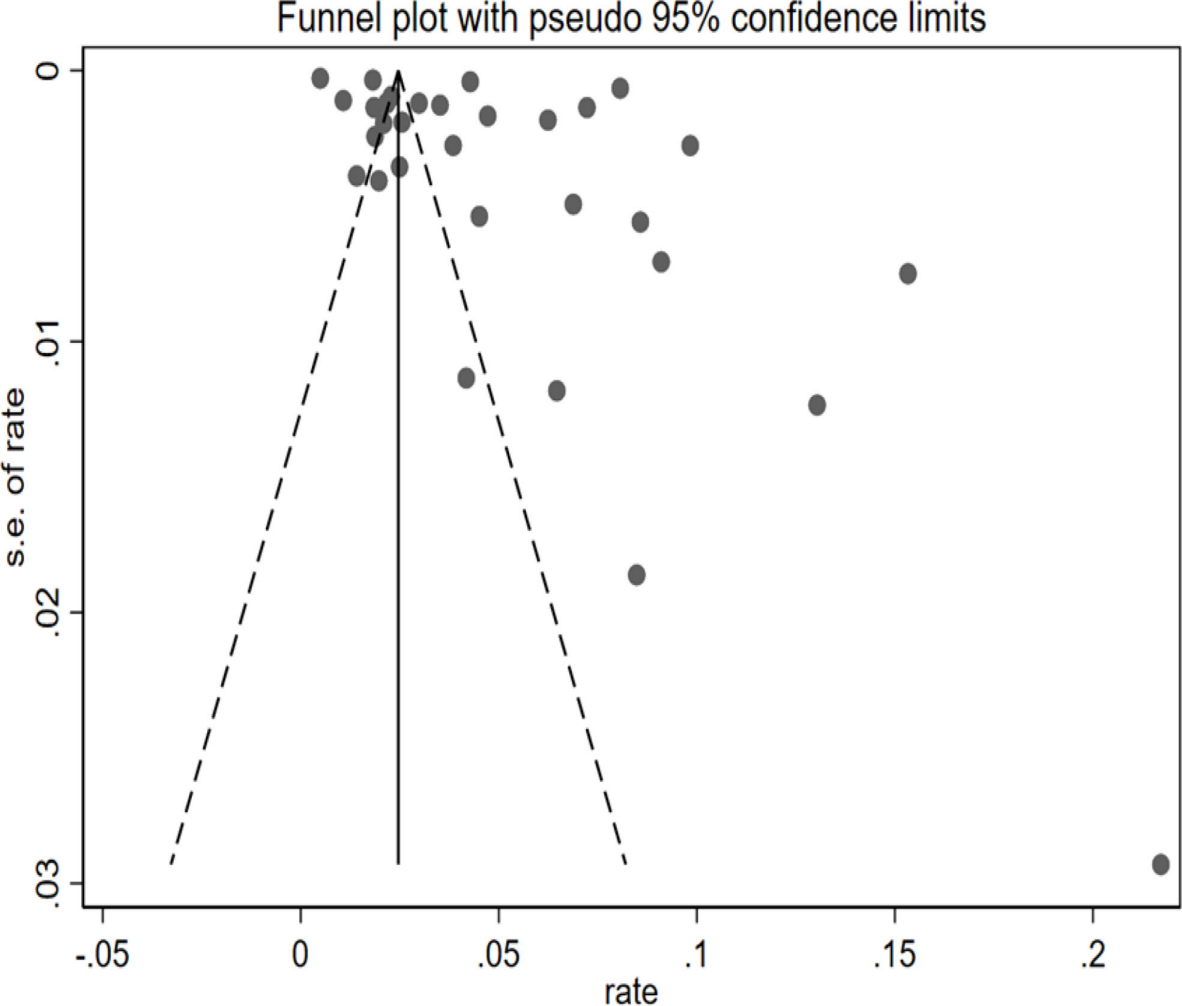
Funnel plot showing the effect of lung cancer in COPD.

The funnel plot exhibited visual asymmetry, whereas Egger’s test regression values (*P* = 0.052) indicated that the difference was insignificant in **Figure 3**. Regression tests indicated no publication bias in this meta-analysis.

### Risk factors for lung cancer in COPD

Four studies (24, 44, 48, 53) reported the sex of patients with COPD and lung cancer, and the pooled OR suggested that sex was not a risk factor for lung cancer in COPD. Smoking status was examined in six studies (24–26, 39, 48, 49), and the analysis results indicated that smoking status of any type did increase the risk of lung cancer, with current smokers showing a higher risk (*P* ≤ 0.05). Five studies (24, 26, 40, 42, 48) focused on the COPD severity as a risk factor for lung cancer, and the results (pooled OR) showed that the risk was statistically significant in patients with mild and moderate COPD (**Table 5**).

**Table 5:** Analysis of the risk factors of lung cancer in COPD.

Risk factors	Studies	Model	OR	Heterogeneity		*P _difference_*
	*n*		(95% CI)	*I* ^2^	*P*	
Gender						
Male	4	Random	0.48 (0.09, 2.66)	99.50%	0	0.398
Female	2	Random	0.13 (0.00, 4.86)	99.70%	0	0.268
COPD severity						
Mild	3	Fixed	1.79(1.23, 2.60)	21.90%	0.278	0.002
Moderate	3	Fixed	2.14(1.44, 3.18)	0	0.931	0.000
Severe	2	Fixed	1.36(0.80, 2.31)	0	0.419	0.251
Very severe	1	Fixed	0.60(0.18, 1.98)	0	0.569	0.404
Smoking status						
Never smoking	3	Fixed	2.94(2.38, 3.64)	31.40%	0.233	0.000
Former smoking	4	Random	3.17(1.30, 7.74)	91.10%	0	0.011
Current smoking	5	Random	3.94(1.28, 12.12)	95.10%	0	0.017

## Discussion

Our review synthesized the current evidence on the prevalence of lung cancer in COPD in 31 populational-based studies covering 829,490 individuals with COPD to show a pooled prevalence of 5.08%, which indicated that lung cancer was an important comorbidity in patients with COPD. Our comprehensive review found that COPD was associated with an increased risk of lung cancer, which is consistent with the findings of previous studies (56, 57). A meta-analysis of a cohort study performed by Zhang et al. (56) showed that the prevalence of lung cancer in patients with COPD was 2.06%, and its subgroup analysis also revealed that locations and COPD severity played a role in increasing the risk of lung cancer. However, their results showed a lower prevalence than ours, which may be attributed to the fact that five (58–62) studies of lung cancer mortality in patients with COPD were included in their analysis, which may have affected the accuracy of the conclusion, particularly underestimating the prevalence of lung cancer in patients with COPD. A population-based review reported that patients with COPD were 6.35 times more likely to have lung cancer than those without COPD, and the pooled prevalence of lung cancer in patients with COPD was 2.79%, which was somewhat different from our results (57). The reason may be related to the different search databases, inclusion and exclusion criteria, and sample size. Unfortunately, their study did not include subgroup analysis or sensitivity analyses, which were adopted in ours to explore the sources of heterogenicity and to confirm that the results had a reliable stability. Furthermore, we pooled the analyses on the risk factors of lung cancer in COPD in order to provide stronger evidence for the relationship between COPD and lung cancer, with the aim of improved prevention and disease management.

The prevalence in male was evidently higher than that in female patients, which is different from the study of Zhang et al. (56). The reason may be that the pooled analysis of a previous systematic review included two studies on lung cancer mortality in COPD, which strikingly affected the analysis results. Also, compared with former smokers, the prevalence of current smokers clearly increased, whereas never smokers with COPD had an exceedingly low risk of lung cancer, indicating that to quit smoking was necessary in patients with COPD. The prevalence was closely related to the severity of COPD (63, 64), and the increased lung cancer risk was 20% when FEV_1_% predicted was decreased by 10% (65). However, our analysis of patients with very severe COPD showed that the prevalence of lung cancer was statistically insignificant, which was mainly attributed to insufficient sample size and demographics discrepancy. The histological subtype showed that adenocarcinoma was the most common cancer in patients with COPD, followed by squamous cell carcinoma, whereas the probability of small-cell occurrence was lower, which was consistent with a previous study (66). The prevalence of lung cancer in COPD was higher in the Western Pacific region than in the European and the Americas regions, which showed similar prevalence. These differences may owe to the relatively backward economic development as well as different aging population and medical conditions in the Western Pacific region.

Understanding the risk factors of lung cancer in patients with COPD can facilitate early prevention and management, thereby reducing the risk of lung cancer. Our result proved that sex should not be interpreted as a risk factor for lung cancer in patients with COPD, which may be associated with increasing female smoking, passive smoking, and indoor air pollutants such as the use of biomass fuel, cooking fumes, as well as poor ventilation systems (67, 68). As in other recent epidemiologic studies (25, 69, 70), the most common risk factor in our study was current smoking, followed by former smoking, and never smoking, which further verifies the harmful effects of tobacco. Also, COPD severity was a common risk factor for lung cancer. In this regard, mild and moderate COPD were statistically significant, which was principally attributed to different demographic characteristics, investigation period, study site, data extraction and processing methods.

The underlying mechanisms of lung cancer predisposition in patients could be deduced and explained based on the characteristics of COPD. First, the inflammatory microenvironment occurring in COPD may increase the probability of DNA damage and mutations (71, 72). Second, some susceptible genes related to COPD can affect the immune microenvironment of the lung by changing their expression pattern in various immune cells, which may lead in turn to the occurrence of lung cancer in COPD (73–75). Third, matrix metalloproteinases not only affect the progression of COPD but also degrade elastic fibers and may thus contribute to the progression and invasion of lung cancer (76, 77). Fourth, tissue hypoxia caused by obstruction of small airways and alveolar capillaries activates hypoxia-inducible factor 1, which can cause tumorigenesis, angiogenesis, and cell multiplication, and therefore accompany a metastatic phenotype (78). In summary, the pathological mechanism of lung cancer in COPD is complex and is related to genetic susceptibility, environmental factors, epithelial-mesenchymal transformation, endothelial-mesenchymal transformation, and extracellular matrix components and functions.

To the best of our knowledge, this systematic review is the largest and most comprehensive of its kind on lung cancer prevalence in patients with COPD. Subgroup and sensitivity analyses were performed to confirm the stability of results. In addition, the quality assessment of most included studies was better, which may have strengthened the reliability of the analysis results. Despite its strengths, our meta-analysis also has several limitations. First, owning to the differences in investigation periods, locations, sample sizes, and demographic characteristics, the heterogeneity of the pooled data was high, which could not be solved even by subgroup analysis. Furthermore, incomplete and missing reports on sex, smoking status, COPD severity, and other variables in the included studies caused imperfect comparisons of all influencing factors. Therefore, positive results should be interpreted with caution.

## Conclusions

This review revealed that the prevalence of lung cancer in patients with COPD is higher, which was supported by evidence-based studies. These findings help to further promote the attention and prevention of lung cancer in patients with COPD and contribute to the development of global management strategies to reduce the occurrence of lung cancer in COPD.

## Data availability statement

The original contributions presented in the study are included in the article/**Supplementary Material**. Further inquiries can be directed to the corresponding author.

## Author contributions

JL and XL contributed to the conception and design of the article; GZ and XL formulated the retrieval strategy and conducted the literature search. GZ, XL, SL, HuZ, and HaZ would answer for data interpretation and analysis; GZ and XL drafted the manuscript; XL, SL, HuZ, HaZ, and JL read and revised it. All authors reviewed and approved the final version of the manuscript.

## Funding

This research was supported by the Chinese Medicine Inheritance and Innovation “Hundred and Ten Million” Talent Project – Chief Scientist of Qi-Huang Project ([2020] No. 219); Zhong-yuan Scholars and Scientists Project (No. 2018204); Characteristic backbone discipline construction project of Henan Province (STG-ZYXKY-2020007).

## Conflict of interest

The authors declare that the research was conducted in the absence of any commercial or financial relationships that could be construed as a potential conflict of interest.

## Publisher’s note

All claims expressed in this article are solely those of the authors and do not necessarily represent those of their affiliated organizations, or those of the publisher, the editors and the reviewers. Any product that may be evaluated in this article, or claim that may be made by its manufacturer, is not guaranteed or endorsed by the publisher.

## Supplementary material

Appendix 1 Search strategy. The retrieval strategies and steps for searching PubMed, EMBASE, Web of Science, and Cochrane Library.

Appendix 2 PRISMA checklist.PRISMA checklist was adopted to normalize the report of this overview, in which the page numbers of the content were detailed.
