# Supplementary material for: Prevalence of lung cancer in chronic obstructive pulmonary disease: A systematic review and meta-analysis
Source: Front Oncol. 2022 Sep 16;12:947981. doi: 10.3389/fonc.2022.947981 (PMC9523743; doi:10.3389/fonc.2022.947981)
Supplement: Supplementary file 2 [file Table_1.docx]

**Supplementary table 1**

**Details of the Literature Search Strategy**

(1) PubMed

| **Search** | **Query** | **Items found** |
| --- | --- | --- |
| #1 | "Pulmonary Disease, Chronic Obstructive"[Mesh] Sort by: Most Recent | 62441 |
| #2 | ((((((Pulmonary Disease, Chronic Obstructive[Title/Abstract]) OR (Chronic Obstructive Pulmonary Disease*[Title/Abstract])) OR (Chronic Obstructive lung Disease[Title/Abstract])) OR (COPD[Title/Abstract])) OR (COAD[Title/Abstract])) OR (Chronic Obstructive Airway Disease[Title/Abstract])) OR (Chronic Airflow Obstruction*[Title/Abstract]) | 76996 |
| #3 | #1 or #2 | 98061 |
| #4 | "Lung Neoplasms"[Mesh] Sort by: Most Recent | 257231 |
| #5 | ((((((lung neoplasm*[Title/Abstract]) OR (lung cancer[Title/Abstract])) OR (pulmonary cancer[Title/Abstract])) OR (lung tumor[Title/Abstract])) OR (pulmonary tumor[Title/Abstract])) OR (lung carcinoma[Title/Abstract])) OR (pulmonary neoplasm*[Title/Abstract]) | 206030 |
| #6 | #4 or #5 | 321133 |
| #7 | #3 AND #6 | 4976 |

(2) Embase

| **Search** | **Query** | **Items found** |
| --- | --- | --- |
| #1 | 'chronic obstructive lung disease'/mj | 76854 |
| #2 | 'chronic obstructive pulmonary disease*':ti OR 'chronic obstructive lung disease':ti OR copd:ti OR coad:ti OR 'chronic obstructive airway disease':ti OR 'chronic airflow obstruction*':ti | 69858 |
| #3 | #1 or #2 | 88432 |
| #4 | 'lung cancer'/mj | 83773 |
| #5 | 'lung cancer':ti OR 'lung carcinoma':ti OR 'lung tumor':ti | 171829 |
| #6 | #4 OR #5 | 198561 |
| #7 | #3 and #6 | 1346 |

(3) Cochrane Library

| **Search** | **Query** | **Items found** |
| --- | --- | --- |
| #1 | MeSH descriptor: [Pulmonary Disease, Chronic Obstructive] this term only | 5860 |
| #2 | (Chronic Obstructive Pulmonary Disease*):ti,ab,kw OR (Chronic Obstructive lung Disease):ti,ab,kw OR (COPD):ti,ab,kw OR (COAD):ti,ab,kw OR (Chronic Obstructive Airway Disease):ti,ab,kw | 22969 |
| #3 | (Chronic Airflow Obstruction*):ti,ab,kw | 606 |
| #4 | #1 or #2 or #3 | 23043 |
| #5 | MeSH descriptor: [Lung Neoplasms] this term only | 7743 |
| #6 | (lung neoplasm*):ti,ab,kw OR (lung cancer):ti,ab,kw OR (pulmonary cancer):ti,ab,kw OR (lung tumor):ti,ab,kw OR (pulmonary tumor):ti,ab,kw | 31583 |
| #7 | (lung carcinoma):ti,ab,kw OR (pulmonary neoplasm*):ti,ab,kw | 10771 |
| #8 | #5 or #6 or #7 | 32134 |
| #9 | #4 and #8 | 1006 |

(4) [Web of Science](https://apps.webofknowledge.com/home.do?SID=6BQQjiiMCVa9MgFvRpC)

| **Search** | **Query** | **Items found** |
| --- | --- | --- |
| #1 | Topic: (**Chronic Obstructive Pulmonary Disease***) OR Topic: (**Chronic Obstructive lung Disease**) OR Topic: (**COPD**) OR Topic: (**COAD**) OR Topic: (**Chronic Obstructive Airway Disease**) OR Topic: **Chronic Airflow Obstruction***) | 52302 |
| #2 | Topic: (**lung neoplasm***) OR Topic: (**lung cancer**) OR Topic: (**pulmonary cancer**) OR Topic: (**lung tumor**) OR Topic: (**pulmonary tumor**) OR Topic: (**lung carcinoma**) OR Topic: (**pulmonary neoplasm***) | 151384 |
| #3 | #1 AND #2 | 926 |
